# Squamous cell carcinoma of the lung showing a ground glass nodule on high-resolution computed tomography associated with pneumoconiosis: a case report

**DOI:** 10.1186/s40792-017-0384-1

**Published:** 2017-09-29

**Authors:** Yuriko Terada, Masaaki Sato, Hiroyuki Abe, Hideki Kuwano, Kazuhiro Nagayama, Jun-ichi Nitadori, Masaki Anraku, Jun Nakajima

**Affiliations:** 1 Department of Thoracic Surgery, The University of Tokyo Graduate School of Medicine, 7-3-1 Hongo, Bunkyo-ku, Tokyo, 113-8655 Japan; 2 Department of Pathology, The University of Tokyo Graduate School of Medicine, 7-3-1 Hongo, Bunkyo-ku, Tokyo, 113-8655 Japan

**Keywords:** Lung cancer, Squamous cell carcinoma, Ground glass nodule, Lepidic growth, Pneumoconiosis

## Abstract

**Background:**

Adenocarcinoma with lepidic growth pattern presents as a ground glass nodule (GGN) on high resolution computed tomography (CT), whereas peripheral pulmonary squamous cell carcinoma (SCC) usually presents as a solid nodule. We herein report a rare case of pulmonary SCC extending along the alveolar lumen representing as a GGN on a CT scan in a patient with pneumoconiosis.

**Case presentation:**

A 77-year-old man with pneumoconiosis was found to have a gradually enlarging GGN in the right lower lobe of the lung on CT. An adenocarcinoma of the lung was suspected. The GGN was successfully resected by thoracoscopic segmentectomy. Pathological examination of the resected specimen was pathologically diagnosed as a stage IA SCC extending along the alveolar lumen. The patient had no evidence of recurrence 19 months after surgery.

**Conclusions:**

SCC should be included in the differential diagnosis of peripherally located GGNs, especially in patients at high risk of SCC of the lung such as those with pneumoconiosis.

## Background

The widespread use of computed tomography (CT) has resulted in increasingly frequent identification of pulmonary ground glass nodules (GGNs). Adenocarcinoma with lepidic growth pattern presents as a GGN on high resolution CT, whereas peripheral pulmonary squamous cell carcinoma (SCC) usually presents as a solid nodule. We herein report a rare case of pulmonary SCC spreading along the alveolar lumen appearing as a GGN on high resolution CT images in a patient with pneumoconiosis.

## Case presentation

**Fig. 1:**
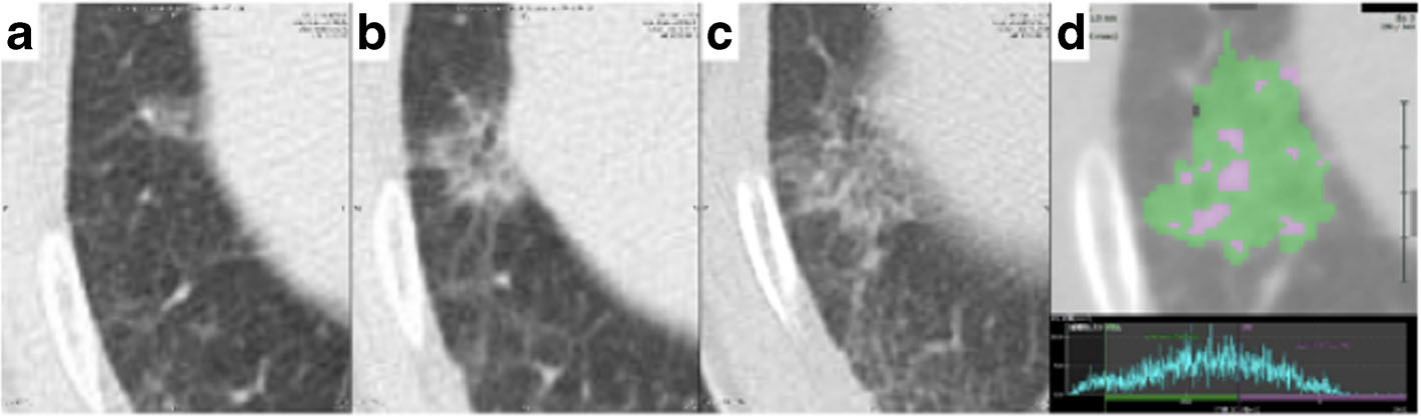
A series of computed tomography images of a squamous cell carcinoma with ground glass nodule. **a** Chest computed tomography (CT) image showing a 7-mm ground glass nodule (GGN) in the *right lower* lobe when the lesion was first noted. **b** Chest CT, obtained 1 year after **a**; the GGN has enlarged to 18 mm. **c** Chest CT image obtained 1.5 year after **a**; the GGN has enlarged to 24 mm and a solid component has developed. **d** In a semi-automated three-dimensional volumetric “GGN analysis” of **c**, the ground glass opacity component (*green area*, between −800 and −301 Hounsfield units) accounted for 64% of the tumor in contrast to the remaining solid component (*purple area*, ≧ −300 Hounsfield units)

**Fig. 2:**
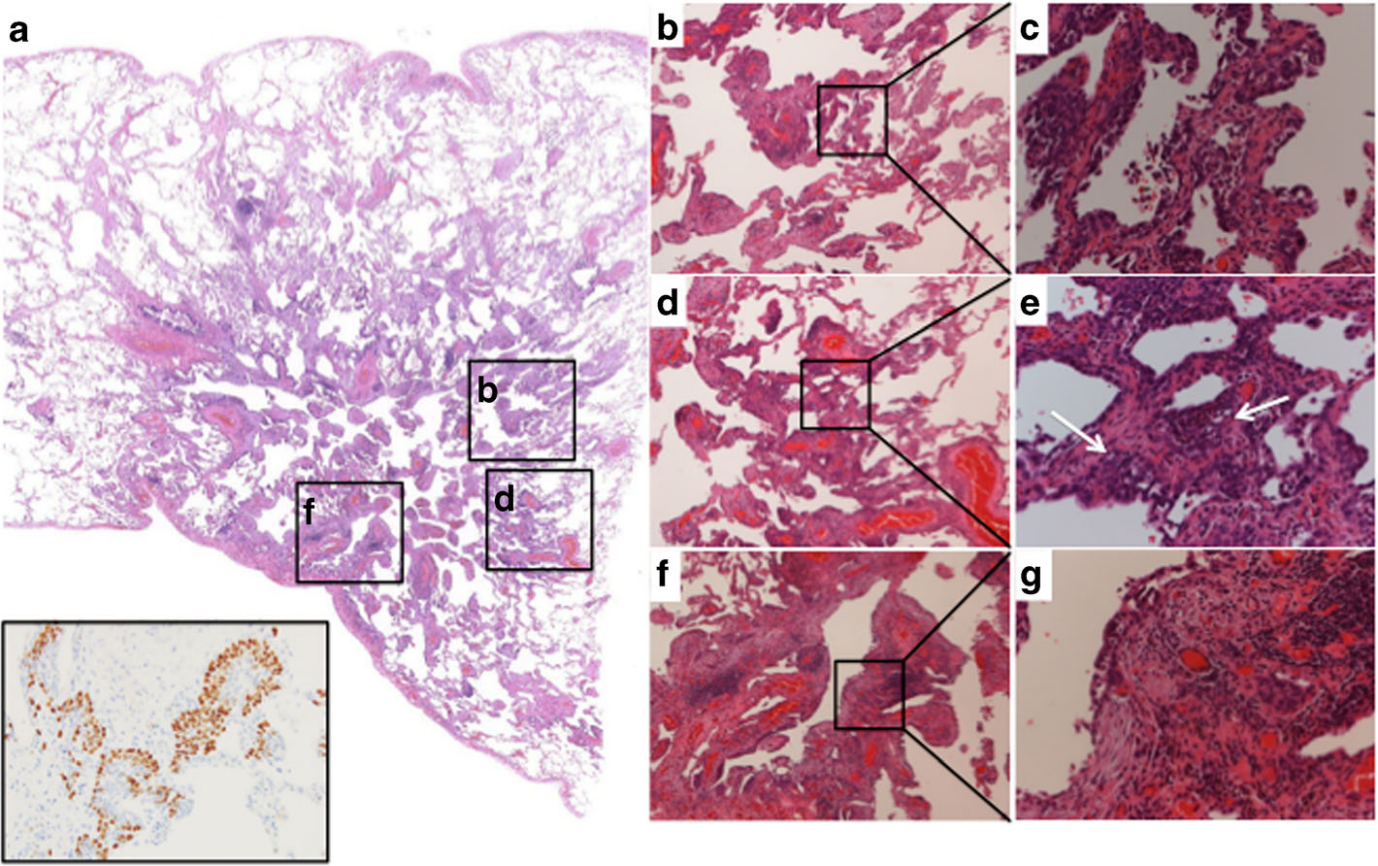
Histopathological findings of the resected specimen. **a** The loupe image of the tumor. The inset shows an immunohistochemistry for p40 (×200), which was positive in the tumor cells. **b** A low-power view (×40) and **c** a high-power view (×200) of the area of carcinoma in situ. Tumor cells were spread along the alveolar wall. The basement membrane of the alveolar wall remained intact. **d** A low-power view (×40) and (**e**) a high-power view (×200) of the area in which carcinoma in situ and invasive carcinoma (arrows) coexisted. **f** A low-power view (×40) and **g** a high-power view (×200) of the area with invasive carcinoma. Stromal tumor cell invasion and stromal collagen fiber were observed. All the pictures are Hematoxylin and eosin stain except the immunohistochemical staining in the inset of **a**

A 77-year-old man with a history of smoking 35 pack-years was found to have a pure GGN in the right lower lobe of the lung on CT during follow-up for pneumoconiosis (Fig. 1a). He had been a potter when he was in his twenties. The GGN gradually enlarged from 0.7 to 2.4 cm over 1.5 years and a solid component developed within it (Fig. 1). In a semi-automated three-dimensional volumetric measurement of ground glass opacity component on the last CT images using a work station, Synapse Vincent© (Fujifilm Medical, Inc., Tokyo Japan), the ground-glass opacity component accounted for 64% of the lesion (Fig. 1d). Laboratory investigations showed normal CEA (3.2 ng/mL; normal value, < 5 ng/mL) and slightly increased SCC antigen (6 ng/mL; normal < 1.5 ng/mL). Lung function studies showed obstructive changes with a vital capacity of 106% and a forced expiratory volume in 1 s/forced vital capacity of 62%. An adenocarcinoma of the lung with a lepidic growth pattern presenting as a GGN with a clinical stage of T1aN0M0 stage IA (UICC-TNM classification, version 7) was suspected. Because of the patient’s limited pulmonary function and relatively non-invasive appearance of the suspected ground-glass-dominant adenocarcinoma lesion [1], a thoracoscopic right S8 + S9b segmentectomy was successfully performed. Histopathological examination of the resected specimen revealed a moderately differentiated SCC of the lung located in S8. Tumor cells with condensed chromatin staining and nuclear enlargement had spread along the alveolar lumen (Fig. 2b, c), whereas stromal tumor cell invasion and stromal collagen fiber were observed (Fig. 2f, g). Keratinization was not apparent. No lymph node metastases or lymphovascular invasion were identified. Fibrosis, silicotic nodules and macules with focal emphysema characteristic of pneumoconiosis were seen in the background lung. The histology was further confirmed by the immunohistochemistry for p40 (the inset of Fig. 2a), a marker for squamous cell carcinoma. The pathological diagnosis was T1aN0M0 stage IA SCC. At 19 months follow-up, there was no evidence of recurrence.

## Discussion

We herein report a peripheral SCC of the lung that presented as a GGN on CT in a patient with pneumoconiosis. Whereas pulmonary adenocarcinoma commonly presents as a GGN, SCC rarely does. The clinical behavior of this variation and appropriate therapeutic strategies remain to be explored.

To our knowledge, four patients with SCC presenting as GGNs have been reported [2–5]. Peripheral pulmonary SCC most commonly progress by filling alveoli, usually resulting in a well-defined solid nodule in CT images; however, SCC do reportedly rarely spread along the alveolar lumen [6, 7] in a similar manner to the progression of adenocarcinoma in situ. In contrast to adenocarcinomas in situ*,* which characteristically replace the normal alveolar lining cells, SCC that spread along the alveolar lumen generally form multilayers of tumor cells between the non-neoplastic alveolar epithelial cells and basement membrane [7, 8]. Consistent with previous reports, the tumor cells had spread along the alveolar lumen in the present case (Fig. 1).

The appearance of SCC presenting as GGNs is similar to that of adenocarcinomas presenting as GGNs on CT images. In adenocarcinomas presenting as GGNs, the nodules become larger and their solid components increase over time. The last CT images before operation still showed 64% of the ground glass opacity component in a three-dimensional volumetric analysis [9]. Histologically, the solid components of these GGNs demonstrate stromal invasion, collapsed alveoli, fibrosis, and masses of tumor cells or macrophages that fill the alveolar sacs, whereas in the areas of pure ground glass appearance tumor cells are spread along the alveolar lumen [10]. In the present case, the tumor initially presented as a pure GGN and gradually developed a solid component; the pathological findings were in agreement with these CT findings (Fig. 2). Air-containing spaces or a bubble-like appearance were reportedly noted in one SCC presenting as a GGN, this phenomenon being characteristic of adenocarcinomas presenting as GGNs [2]. In these respects, SCC and adenocarcinoma presenting as GGNs appear similar on CT scans.

Unlike the previously reported cases of SCC presenting as GGNs, the present case is unique in that the patient had underlying pneumoconiosis. In the background lung, fibrosis, silicotic nodules, and macules which are characteristic of pneumoconiosis were identified. Also, focal emphysema was found in the background lung which seemed to secondary to smoking or pneumoconiosis. Pneumoconiosis increases the risk of lung cancer, SCC being the most frequent type of associated lung cancer [11]. Inhalation of carcinogens may play a role in the pathogenesis of lung cancer [12]. It has been reported that SCC arising in patients with pneumoconiosis are significantly more frequently of the peripheral type than those in patients without pneumoconiosis. Peripheral-type SCC also arise preferentially in the lower lobes of patients with pneumoconiosis [11]. In the present case, given that the histologic tumor type was SCC and the tumor arose from a lower lobe, pneumoconiosis may have contributed to its development. SCC should be included in the differential diagnosis of peripherally located GGNs, especially in patients at high risk of SCC of the lung such as those with pneumoconiosis.

Surgical resection, comprising one segmentectomy and three lobectomies, was performed in all four previously reported patients with SCC presenting as GGNs [2–5]. All these lesions were at an early stage and had good prognoses. In the present case, because the patient had impaired pulmonary function and the tumor was believed to be a ground-glass-dominant adenocarcinoma, we performed segmentectomy. Although sublobar resection is reportedly appropriate in selected patients with such tumors because they are rarely invasive and rarely have lymph node metastases [1], whether sublobar resection for peripheral SCC appearing as GGNs is adequate is unknown. The volume doubling time of the tumor in the present case was about 100 days, whereas the reported volume doubling times of part-solid GGNs that prove to be adenocarcinomas are 276.9–1228.5 days [13–15]. This may indicate that the volume doubling times of SCC presenting as GGNs are shorter than those of such adenocarcinomas and that such SCC are potentially more aggressive than those adenocarcinomas. However, there are too few reported cases of SCC presenting as GGNs to draw definite conclusions; more studies are needed to assess their clinicopathological features and determine the most appropriate therapeutic strategies for them.

## Conclusions

In summary, we here report a rare case of pulmonary SCC presenting as a GGN on high resolution CT in a patient with pneumoconiosis, which is a risk factor for peripheral pulmonary SCC. We highlight that the differential diagnosis of peripherally located GGNs should include an SCC extending along the alveolar lumen and that these tumors may be more aggressive than adenocarcinomas presenting as GGNs.

## Abbreviations

CT: Computed tomography
GGN: Ground glass nodule
SCC: Squamous cell carcinoma
